# Potential of *Lactobacillus plantarum* CCFM639 in Protecting against Aluminum Toxicity Mediated by Intestinal Barrier Function and Oxidative Stress

**DOI:** 10.3390/nu8120783

**Published:** 2016-12-02

**Authors:** Leilei Yu, Qixiao Zhai, Fengwei Tian, Xiaoming Liu, Gang Wang, Jianxin Zhao, Hao Zhang, Arjan Narbad, Wei Chen

**Affiliations:** 1 State Key Laboratory of Food Science and Technology, School of Food Science and Technology, Jiangnan University, Wuxi 214122, China; edyulei@126.com (L.Y.); zhaiqixiao@sina.com (Q.Z.); fwtian@jiangnan.edu.cn (F.T.); liuxm@jiangnan.edu.cn (X.L.); wanggang@jiangnan.edu.cn (G.W.); jxzhao@jiangnan.edu.cn (J.Z.); zhanghao@jiangnan.edu.cn (H.Z.); 2 UK-China Joint Centre on Probiotic Bacteria, Norwich NR4 7UA, UK; 3 Gut Health and Food Safety Programme, Institute of Food Research, Norwich NR4 7UA, UK; 4 Beijing Innovation Centre of Food Nutrition and Human Health, Beijing Technology & Business University, Beijing 100048, China

**Keywords:** probiotic, lactic acid bacteria, *Lactobacillus plantarum*, aluminum, protection, gut health

## Abstract

Aluminum (Al) is a ubiquitous metal that can seriously harm the health of animals and humans. In our previous study, we demonstrated that *Lactobacillus plantarum* CCFM639 can decrease Al burden in the tissues of mice by inhibiting intestinal Al absorption. The main aim of the present research was to investigate whether the protection by the strain is also associated with enhancement of the intestinal barrier, alleviation of oxidative stress and modulation of the inflammatory response. In an in vitro cell model, two protection modes (intervention and therapy) were examined and the results indicated that *L. plantarum* CCFM639 alleviated Al-induced cytotoxicity. In a mouse model, *L. plantarum* CCFM639 treatment was found to significantly alleviate oxidative stress in the intestinal tract, regulate the function of the intestinal mucosal immune system, restore the integrity of tight junction proteins and maintain intestinal permeability. These results suggest that in addition to Al sequestration, *L. plantarum* CCFM639 can also inhibit Al absorption by protecting the intestinal barrier, alleviating Al-induced oxidative stress and inflammatory response. Therefore, *L. plantarum* CCFM639 has the potential to be a dietary supplement ingredient that provides protection against Al-induced gut injury.

## 1. Introduction

Aluminum (Al) is the most abundant metal in nature and is widely used in daily life [1]. The principal exposure route to Al for humans is through food, water and beverages [2]. The mean levels of Al exposure via this route range from 0.2 to 1.5 mg/kg of body weight (bw) weekly in Europe and 1.3 mg/kg·bw/week for adults and 3.3 mg/kg·bw/week for children in China [3]. The European Food Safety Authority (EFSA) established a health-based guidance value for 1 mg/kg·bw/week, which is thus exceeded in China [4]. The excess Al could accumulate in most of the tissues, including brain, liver, kidneys, spleen and bone, and may cause Alzheimer’s disease, dialysis encephalopathy, microcytic anemia, and osteomalacia [5]. The intestinal tract is the first barrier against Al exposure. Previous studies reported that the intestinal tract was the main storage organ for Al: forty percent of the ingested Al was found to accumulate in the intestinal mucosa [6,7]. The intestinal barrier consists of a single layer of epithelial cells and tight junction (TJ) proteins, acting as the body’s first line of defense against harmful substances, including heavy metals [8]. Once Al is absorbed, it can induce oxidative stress and intestinal inflammation [2,9]. At the same time, the absorbed Al can damage TJ proteins and impair the intestinal barrier function and cause cell apoptosis, thereby increasing the intestinal permeability [2].

Probiotics exhibit a variety of health-promoting functions [10] and there is accumulating evidence that some probiotics have the potential to alleviate metal toxicity [11,12]. Moreover, probiotics can provide benefits to the host gut through a diverse set of mechanisms that include modulation of immune responses, alleviation of oxidative stress and reduce intestinal permeability by maintenance of intestinal barrier integrity by expression and distribution of TJ proteins [13,14,15]. TJ proteins comprise a large group of proteins, including the scaffolding proteins zonula occludens-1 (ZO-1), and the transmembrane proteins occludin and claudins, which are critical in maintaining barrier function [16]. A number of lactobacilli have been shown to improve intestinal barrier function, partly by modulating the expression and distribution of the TJ proteins [13,14].

Our previous studies demonstrated the protection against Al toxicity in vitro and in vivo by the probiotic strain *Lactobacillus plantarum* CCFM639 was due to its enhanced Al-binding and antioxidative capabilities [17,18]. It significantly inhibits Al absorption in the intestines of mice, thereby decreasing Al accumulation in tissues and alleviating tissue injury caused by this metal. A number of studies have demonstrated that probiotics can protect intestinal health [19,20]. We hypothesized that *L. plantarum* CCFM639 can inhibit Al absorption by protecting the intestinal integrity and modulating the inflammatory response.

## 2. Materials and Methods

### 2.1. Bacterial Strains and Culture Conditions

*L. plantarum* CCFM639 (CGMCC9664) was obtained from the in-house Culture Collections of Food Microbiology in Jiangnan University (CCFM, Wuxi, China) and stored in the China General Microbiological Culture Collection Center (CGMCC, Beijing, China). It was grown in MRS broth (Qing Dao Hopebio-Technology Co., Ltd., Qingdao, China) at 37 °C.

### 2.2. Cell Culture Experiment

HT-29 cells were obtained from the Chinese Academy of Sciences (Shanghai, China). The cells were grown in RPMI-1640 medium, supplemented with 10% (*v*/*v*) fetal bovine serum (FBS) and 100 U/mL penicillin/streptomycin (PS) at 37 °C in an atmosphere of 5% CO_2_. The media and supplements were purchased from Gibco (New York, NY, USA).

The HT-29 cells were seeded in and cultured in different formats, including 96-well plates, 12-well plates, 6-well plates and 2-cm-diameter cover glass-bottom dishes, at a density of 10^5^ cells/mL, and grown in an atmosphere of 5% CO_2_ at 37 °C. When the cells attached to the plates and formed monolayers, Al ions and the prepared *L. plantarum* CCFM639 were added at final concentrations of 4 mM and 10^8^ CFU/mL, respectively (Table 1). The dose of Al exposure was selected based on a previous study [2] and on the data obtained from our preliminary cell viability assay using 3-(4,5-dimethylthiazol-2-yl)-2,5-diphenyl tetrazolium bromide (MTT) (Figure S1).

Medium, RPMI-1640 supplemented with fetal bovine serum (FBS) and penicillin–streptomycin (PS); Medium + Al, 4 mM Al ion in medium; Medium + 639, 1 × 10^8^ CFU/well *L. plantarum* CCFM639 in medium; Medium + Al + 639, 4 mM Al ion and 1 × 10^8^ CFU/well *L. plantarum* CCFM639 in medium. PW, plain water for drinking; SM, 0.2 mL skim milk; Al, Al ion at 200 mg/L in drinking water; DFP, 0.2 mL DFP (2.5 g·L^−1^) in plain water. SM + 639, 0.2 mL skim milk contained 1 × 10^9^ CFU *L. plantarum* CCFM639 once a day administered via oral gavage. SM + DFP, 0.2 mL DFP administered once a day via oral gavage.

### 2.3. Animals and Experimental Design

Forty adult C57BL6 mice (6-week-old males, 18–25 g) were bought from the Shanghai Laboratory Animal Center (Shanghai, China). The mice were housed in a room with constant temperature and humidity and a 12 h light-dark cycle was maintained. The standard commercial mouse food and deionized water were given ad libitum. All of the protocols were approved by the Ethics Committee of Jiangnan University (Wuxi, China, JN No. 20150312-0717-10). The care procedures for the animals were implemented following the European Community guidelines (directive 2010/63/EU).

The mice were randomly divided into control, Al only, Al plus CCFM639 and Al plus deferiprone (DFP) groups (Table 1). An oral dose of Al ions (200 mg/L) in drinking water was used for establishing the Al exposure model [21,22]. The probiotic strain was given via gavage at a dose of 1 × 10^9^ CFU in 0.2 mL of skim milk once daily. The skim milk was used as a protectant to avoid the loss of viability during lyophilization. The DFP was administered in 0.2 mL at a concentration of 2.5 g/L via oral gavage as a positive control. At the end of the experiment, each mouse was anesthetized by ether and then sacrificed.

### 2.4. Measurement of Cytotoxicity

The cell viability was measured by MTT assay according to the procedure previously described [23]. The assay kit was obtained from the Institute of Beyotime Biotechnology (Jiangsu, China). The result of the cell viability was expressed as the ratio to the level in the control group. Apoptotic rate was measured with an Annexin V-FITC/PI kit (Institute of Beyotime Biotechnology, Jiangsu, China), and the FITC levels were then detected by flow cytometry (FACSCalibur, Becton Dickinson, San Diego, CA, USA) [23].

### 2.5. Measurement of Tight Junction Proteins in HT-29 Cell Line

The morphologies of the TJ proteins were evaluated by immunofluorescence staining as previously described [24]. Firstly, HT-29 cells were fixed, permeabilized and blocked. They were then incubated with the primary antibodies at 1:50 dilution, including anti-ZO-1, anti-claudin-1 and anti-occludin (Life Technologies, Rockford, AL, USA), overnight at 4 °C. The cells were then incubated with secondary antibodies, Alexa Fluor 488-conjugated goat anti-rabbit at 1:50 dilution (Life Technologies, Rockford, AL, USA), for 1 h at room temperature. Finally, the cells were exposed to the nuclear stain 4′,6-diamidino-2-phenylindole (DAPI) for 10 min at room temperature. Images of the stained cells were acquired using confocal laser scanning microscopy (TCS SP8, Leica, Mannheim, Germany).

### 2.6. Measurement of Tight Junction Proteins in Mice Intestinal Tissues

The small intestine and colon sections were prepared by blocking with 5% bovine serum albumin (BSA) (Sigma, Dorset, UK) and 0.5% Triton X-100 (Sigma, Dorset, UK) in PBS. The tissues were incubated with primary antibodies, anti-occludin (Santa, San Fransisco, CA, USA, sc-271842) and anti-claudin-1 (Santa, San Fransisco, CA, USA, sc-166338) and anti-ZO-1 (Abcam, Cambridge, UK, ab59720). They were then incubated with secondary antibodies, goat anti-mouse Alexa 488 (Abcam, Cambridge, UK, ab150113) or goat anti-rabbit Alexa 488 (Abcam, Cambridge, UK, ab150077) for 1 h at room temperature. Nuclear staining and imaging were performed as described above.

The mRNA expressions of the TJ proteins in intestinal tissues were evaluated by real-time quantitative PCR (RT-qPCR). Approximately 0.1 g of small intestine and colon fragments were isolated quickly and preserved in liquid nitrogen immediately. Total RNA was extracted with Trizol reagent (Invitrogen, Carlsbad, CA, USA) following the manufacturer’s operating instructions, and the extracted RNA samples were analyzed by NanoDrop and via gel electrophoresis. The RNA was then transcribed to cDNA using the PrimeScript First Strand cDNA Synthesis Kit (Takara, Tokyo, Japan). Gene expression was investigated using a RT-qPCR system (Bio-Rad, Hercules, CA, USA) and the sequences of the RT-qPCR primers used were as follows [25]: claudin-1 forward (F): 5′-GATGTGGATGGCTGTCATTG-3′ and reverse (R): 5′-CCTGGCCAAATTCATACCTG-3′; occludin F: 5′-CACACTTGCTTGGGACAGAG-3′ and R: 5′-TAGCCATAGCCTCCATAGCC-3′; ZO-1 F: 5′-CTTCTCTTGCTGGCCCTAAAC-3′ and R: 5′-TGGCTTCACTTGAGGTTTCTG-3′; and β-actin F: 5′-GGCTGTATTCCCCTCCATCG-3′ and R: 5′-CCAGTTGGTAACAATGCCATGT-3′.

### 2.7. Measurement of Intestinal Permeability of Mice

The intestinal permeability of the mice was analyzed by endotoxin assay as described in a previous study [26]. After sacrifice, the endotoxin levels in the serum were detected using an ELISA kit (Abcam, Cambridge, UK).

### 2.8. Measurement of Oxidative-Stress-Related Parameters in HT-29 Cell Cultures and Serums

The activities of superoxide dismutase (SOD) and catalase (CAT) and the level of malondialdehyde (MDA) were used to evaluate oxidative stress [17]. The level of MDA and activities of SOD and CAT in HT-29 cells and serums of mice were determined by assay kits (Nanjing Jiancheng Bioengineering, Jiangsu, China). The level of reactive oxygen species (ROS) in HT-29 cells was measured by S0033 assay kit (Institute of Beyotime Biotechnology, Jiangsu, China) following manufacturer’s instructions.

### 2.9. Measurement of Pro-Inflammatory Cytokines in HT-29 Cell Cultures and Intestinal Tissues

The HT-29 cells were centrifuged at 15,000× *g* for 15 min at 4 °C to obtain the supernatant. The small intestine and colon samples were homogenized with 1 mL of radioimmunoprecipitation assay (RIPA) buffer (Beyotime Biotechnology, Jiangsu, China) and then centrifuged under the same conditions to obtain the supernatant [26]. The levels of TNF-α, IL-1β and IL-6 in the supernatant were detected using an ELISA kit (Abcam, Cambridge, UK) following the manufacturer's instructions.

### 2.10. Statistical Analysis

The data were expressed as the mean ± standard error of the mean (SEM). The data were statistically analyzed using SPSS 16.0 (SPSS Inc., Chicago, IL, USA). Differences between the data were analyzed using one-way analysis of variance (ANOVA), followed by Tukey’s post hoc test. Values of *p* < 0.05 were considered to be statistically significant.

## 3. Results

### 3.1. Effects of L. plantarum CCFM639 on Al-Induced Cytotoxicity

The Al ion cytotoxic concentration for HT-29 cells was determined by MTT assay (Figure S1). The result show that the cell viability decreased with the increase of Al ion concentration and reduced to approximately 50% at an Al ion concentration of 4 mM, so this concentration was selected to be used in the subsequent experiments. Compared with the control group, the HT-29 cell viability in the Al-only group decreased significantly (*p* < 0.05, Figure S2B). Treatment with CCFM639 was found to substantially reverse the decrease in cell viability in both intervention assay (treatment simultaneously with Al exposure) and therapy assay (treatment after Al exposure). The flow cytometry data indicate that the percentage of apoptotic cells in the control group was less than 10%, while Al exposure significantly increased the apoptotic cell count to 28.73% in the intervention assay and 32.85% in the therapy assay (Figure S2A,C). Compared with the Al-only group, treatment with the probiotic exhibited a significant protective effect against Al-induced cell apoptosis and the values drop to 17.50% in intervention assay and 19.71% in therapy assay (*p* < 0.05). However, the treatment with CCFM639 only had no marked effect on cell viability or cell apoptosis.

### 3.2. Effects of L. plantarum CCFM639 on Al-Induced Alterations of Tight Junction Proteins

**Figure 1:**
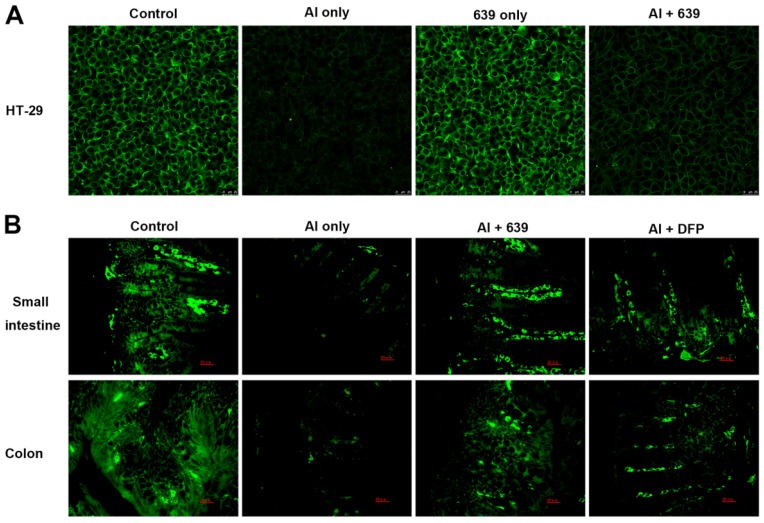
Effects of *L. plantarum* CCFM639 on Al-induced alterations of tight junction protein ZO-1: in vitro (**A**); and in vivo (**B**). The ZO-1 levels in HT-29 cell (therapy assay) and intestine of mice (intervention assay) were observed with confocal microscopy and stained by Alexa Fluor 488 (green).

**Figure 2:**
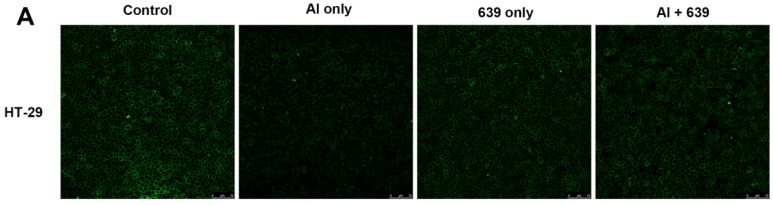

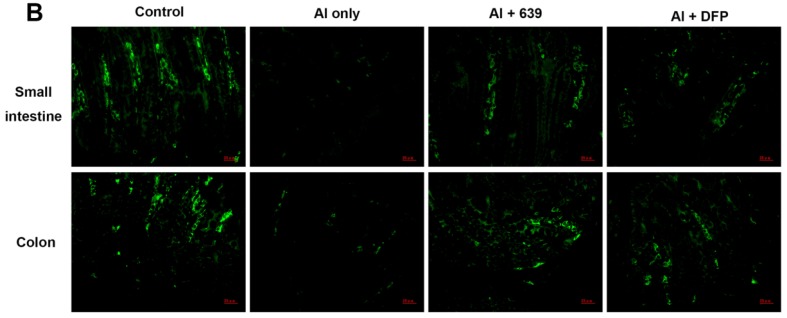
Effects of *L. plantarum* CCFM639 on Al-induced alterations of tight junction protein claudin-1: in vitro (**A**); and in vivo (**B**). The claudin-1 levels in HT-29 cell (therapy assay) and intestine of mice (intervention assay) were observed with confocal microscopy and stained by Alexa Fluor 488 (green).

**Figure 3:**
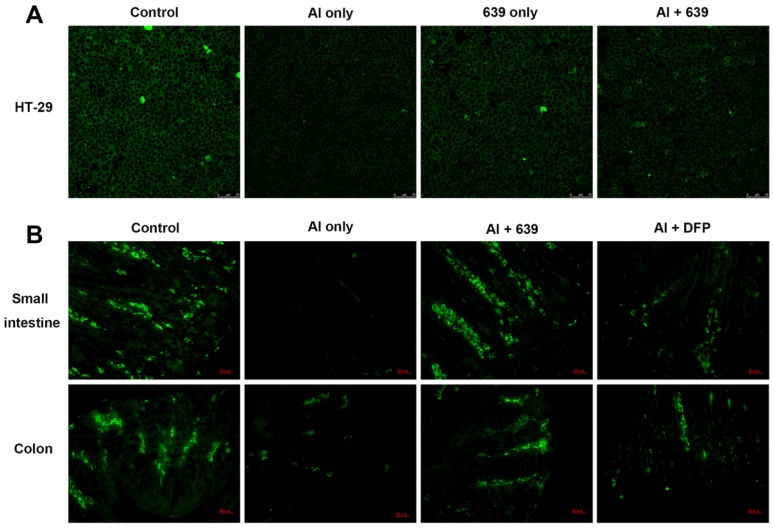
Effects of *L. plantarum* CCFM639 on Al-induced alterations of tight junction protein occludin: in vitro (**A**); and in vivo (**B**). The occludin levels in HT-29 cell (therapy assay) and intestine of mice (intervention assay), were observed with confocal microscopy and stained by Alexa Fluor 488 (green).

The tight junction (TJ) proteins (ZO-1, claudin-1, and occludin) in the cell monolayers were analyzed qualitatively (Figure 1A, Figure 2A and Figure 3A). In the control group, the TJ proteins were continuously distributed along the cell boundaries. After Al exposure, the TJ proteins were discontinuously distributed at sites of cell-to-cell contact. Co-treatment with CCFM639 and Al markedly alleviated the Al-induced damage to the TJ proteins.

**Figure 4:**
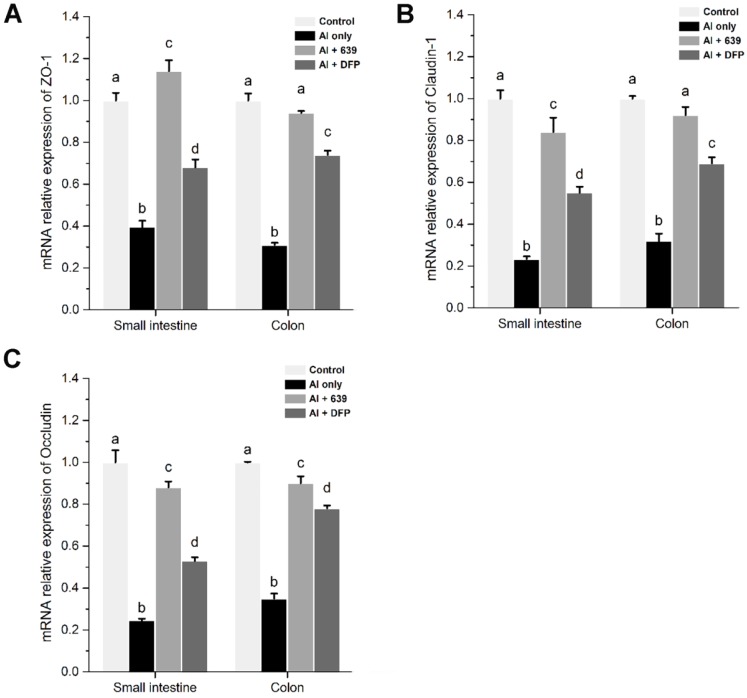
Effects of *L. plantarum* CCFM639 on Al-induced alterations of mRNA expression levels of tight junction proteins in intestine: (**A**) the mRNA expression levels of ZO-1; (**B**) the mRNA expression levels of claudin-1; and (**C**) the mRNA expression levels of occludin. Values are expressed as fold change versus control group and presented as the mean ± SEM. The different letters a–d indicate statistically significant changes among the four groups (*p* < 0.05).

In the in vivo study using the immunofluorescence assay, representative images of TJ proteins staining in the small intestine and colon samples are shown in Figure 1B, Figure 2B and Figure 3B. Exposure to Al resulted in a decrease of staining intensity and density for the three TJ proteins in both the small intestine and colon. Treatment with CCFM639 markedly increased the staining intensities and densities with a lesser effect shown by DFP. Therefore, *L. plantarum* CCFM639 demonstrated a better protective effect against Al-induced damage to the intestinal TJ proteins than DFP. Moreover, Al exposure significantly reduced the mRNA expression of the three TJ proteins in the colons and small intestines of mice (Figure 4C, *p* < 0.05). In contrast to the Al only group, the expression of all of these proteins was partially restored by treatment with CCFM639 or DFP, and indeed CCFM639 increased the three TJ protein mRNA to levels close to the control group.

### 3.3. Effects of L. plantarum CCFM639 on Gut Permeability

**Figure 5:**
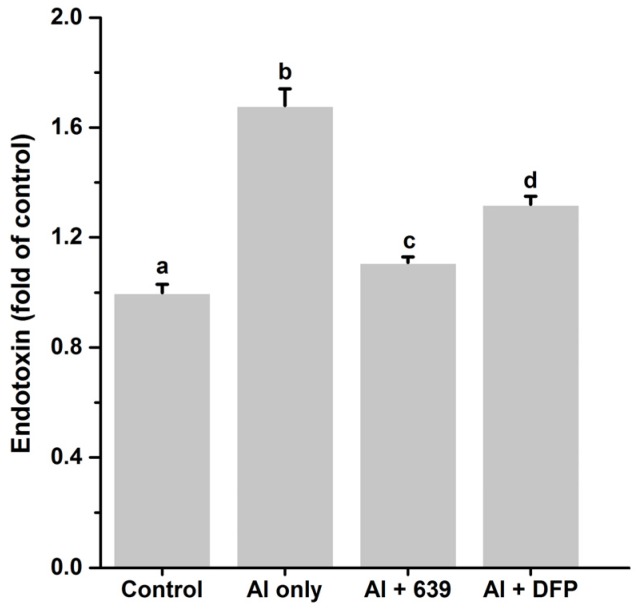
Effects of *L. plantarum* CCFM639 on Al-induced alterations of endotoxin level in serum of mice. Values are expressed as fold change versus control group and presented as the mean ± SEM. The different letters a–d indicate statistically significant changes among four groups (*p* < 0.05).

Endotoxin translocation is related to disruption of the gut barrier [27]. Therefore, the endotoxin level in the serum was used to evaluate the intestinal permeability. As shown in Figure 5, after Al exposure, the endotoxin level markedly increased, indicating that Al exposure was related to the increase of gut permeability. Moreover, ingestion of the probiotic strain markedly reduced gut permeability, and *L. plantarum* CCFM639 had a better protective effect than DFP.

### 3.4. Effects of L. plantarum CCFM639 on Al-Induced Oxidative Stress In Vitro and In Vivo

**Figure 6:**
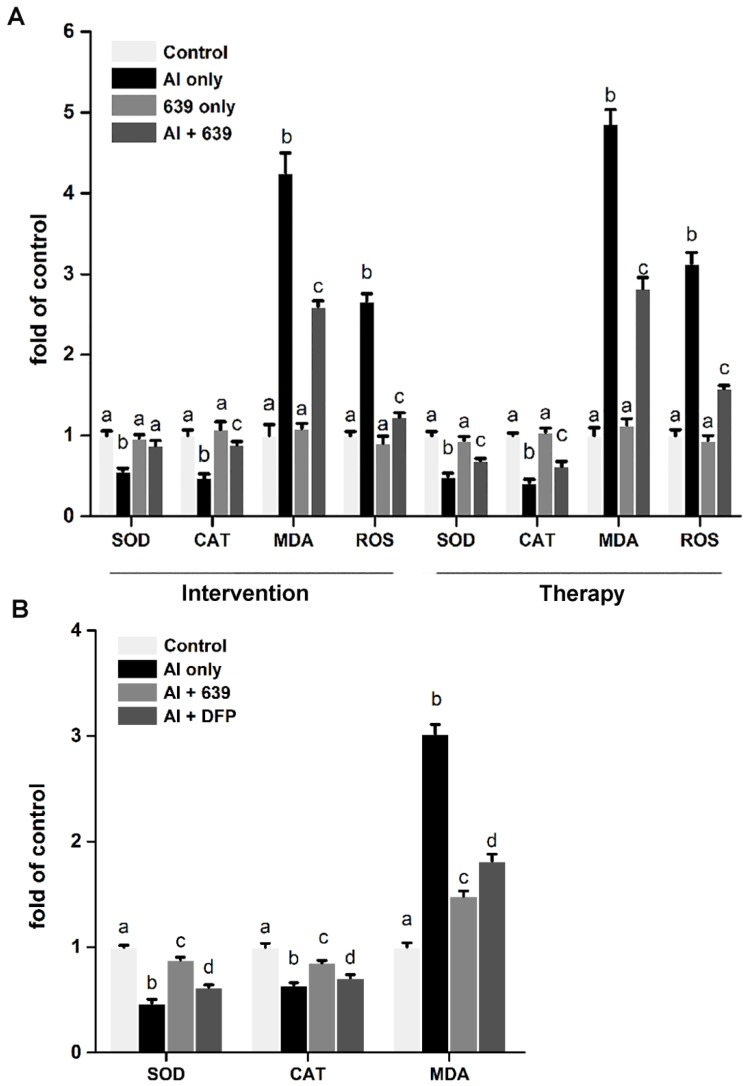
Effects of *L. plantarum* CCFM639 on Al-induced oxidative stress in HT-29 cells and serum of mice: (**A**) the activities of SOD and CAT and levels of MDA and ROS in HT-29 cells; and (**B**) the activities of SOD and CAT and MDA level in serum of mice. Values are expressed as fold change versus control group and presented as the mean ± SEM. The different letters a–d indicate statistically significant changes among four groups (*p* < 0.05).

As shown in Figure 6, both in vitro and in vivo assays, Al exposure markedly increased levels of ROS and MDA and decreased the activities of SOD and CAT. Moreover, in the in vitro assays, the ROS levels in the intervention and therapy assays were enhanced by 2.66 and 3.13 fold, respectively, compared with the control group (Figure 6A). Co-treatment with CCFM639 and Al dramatically inhibited Al-induced ROS generation, reduced the MDA level and increased the activities of SOD and CAT in both the intervention and therapy assays (Figure 6A; *p* < 0.05). The results of ROS were further confirmed by confocal microscopy performed immediately (Figure S3). However, there was no significant change in the levels of ROS or MDA between the control and the CCFM639 only group. In vivo, *L. plantarum* CCFM639 or DFP treatment led to a significant decrease of MDA levels and an increase of SOD and CAT activities in serum of mice (Figure 6B; *p* < 0.05). However, *L. plantarum* CCFM639 had a more pronounced effect than DFP on these oxidative-stress-related parameters.

### 3.5. Effects of L. plantarum CCFM639 on Al-Induced Alterations of Pro-Inflammatory Cytokines In Vitro and In Vivo

Al exposure significantly increased the levels of pro-inflammatory cytokines TNF-α, IL-1β and IL-6 in both HT-29 cells and intestinal tissues (Table 2 and Table 3; *p* < 0.05). In the in vitro assays CCFM639 treatment was found to restore the levels of all three cytokines in both the intervention and therapy assays (Table 2). However, CCFM639 only treatment did not cause significant differences in the levels of the three pro-inflammatory cytokines compared with the control groups.

In vivo, the ingestion of CCFM639 or DFP markedly reversed all of the Al-induced alterations to levels of cytokines, with the exception of IL-6 in the colon (Table 3). Moreover, *L. plantarum* CCFM639 was more efficient at decreasing the impact of Al on these pro-inflammatory cytokines than DFP.

## 4. Discussion

A number of studies have documented the adverse effects of Al on the brain, liver and bone [28,29]. Recently, more attention has been paid to the harmful effects of Al on the intestine, including injury of intestinal barrier, decrease of tight junction (TJ) protein expression and stimulation of intestinal inflammation [2,3]. This study demonstrated that chronic Al exposure can cause the disruption of TJ proteins, increase intestinal permeability and induce oxidative stress and inflammatory response. Moreover, we observed that *L. plantarum* CCFM639 treatment could increase Al excretion [17,18] and protect the intestinal barrier, which acts as the body’s first line of defense against oral Al exposure. Meanwhile, *L. plantarum* CCFM639 has the ability to alleviate oxidative stress and inflammatory response. Therefore, we hypothesized that *L. plantarum* CCFM639 can reduce Al absorption and protect intestinal health possibly via initial intestinal Al sequestration, protection of TJ proteins, alleviation of oxidative stress and inflammatory response.

Moreover, Lactobacilli need to survive exposure to gastric acid and bile before reaching the intestines and this passage can reduce the numbers of viable bacteria. A large number of studies show that oral administration of probiotics should be more than 10^8^ CFU per day, which can permit viable bacteria levels to be more than 10^6^ CFU in human or animal intestines that can then provide the beneficial effects [30,31,32]. Therefore, a dose of 10^9^ CFU was selected in the present study.

**Figure 7:**
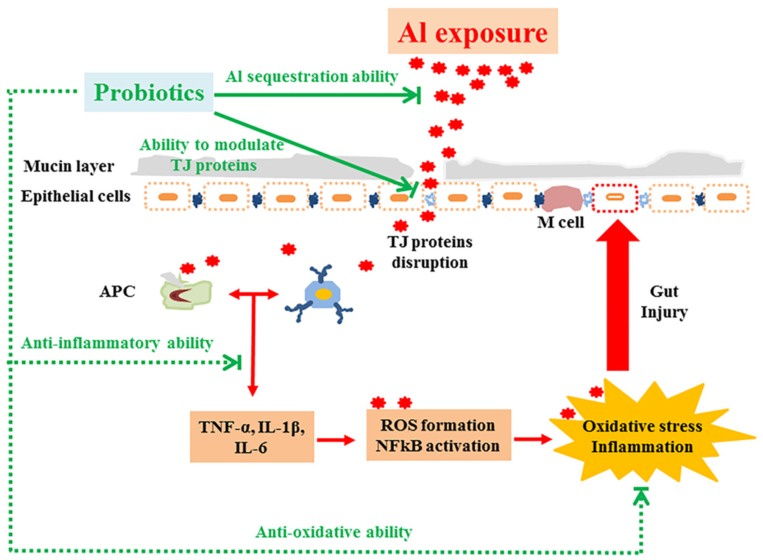
The potential protection mechanism of probiotics against Al-induced gut injuries.

Our previous study found that a probiotic *L. plantarum* CCFM639 could reduce Al absorption in the intestines of mice due to its excellent Al-binding ability [17,18]. The decrease of Al absorption in the intestines in turn protects the intestine from Al-induced injures. It is believed that intestinal absorption of Al includes paracellular and transcellular routes. The former is through TJ proteins by passive processes, and the latter is through the enterocytes involving passive and active transport processes [1,33,34,35]. The results of in vitro therapy assay in this study showed that *L. plantarum* CCFM639 can significantly maintain the vitality of cells and reverse the disruption of TJ proteins even 24 h after Al treatment (the strain had no direct contact with the aluminum). These results indicated that *L. plantarum* CCFM639 can protect against Al toxicity, not only via the Al-binding route but also by other protective routes (Figure 7). Increasing evidence indicates that some probiotics can improve gut barrier dysfunction caused by toxic substances [36,37,38]. Lactobacilli are one of the well-known groups of beneficial members of the intestinal microbiota and very commonly are used in probiotic foods [39]. Previous studies have demonstrated that Lactobacilli could maintain a normal mucosal barrier via modulation of TJ proteins [15,40]. Al exposure damage TJ proteins located between pairs of epithelial cells, which may cause intercellular leakage and promote Al movement across the intestinal barrier [2,41,42]. Moreover, Al induces cell damage and apoptosis, which may lead to leakage in the epithelial layer, causing a greater amount of Al to enter the systemic circulation [42,43]. Therefore, maintaining the integrity of the gut barrier plays a very important role in inhibiting intestinal Al absorption. The results showed that *L. plantarum* CCFM639 can significantly increase TJ proteins levels in vivo. Therefore, modulation of TJ proteins may be one route by which *L. plantarum* CCFM639 protects barrier function (Figure 7).

The mechanisms of Al toxicity also include oxidative stress and inflammatory response [28,29]. Al exposure causes oxidative stress with a decrease in SOD and CAT activities and a higher level of MDA [17]. It also stimulates production of pro-inflammatory cytokines [2], including tumor necrosis factor-α (TNF-α), interleukin-1β (IL-1β) and IL-6. Moreover, pro-inflammatory cytokines can activate the NF-kB pathway, which has been identified to be critical to oxidative stress, and then generate reactive oxygen species (ROS) [44]. At the same time, the production of ROS will aggravate the inflammatory process [11]. Oxidative stress may result in loss of tight junction integrity and increased paracellular permeability [45]. There is increasing evidence indicating that probiotics have beneficial effects on intestinal inflammation [10,46]. They also cause an inhibition of the NF-kB pathway, which thereby alleviates oxidative stress [47,48]. In present study, *L. plantarum* CCFM639 was not in direct contact with Al in the therapy assay, but it still significantly inhibited the generation of ROS and pro-inflammatory cytokines, reduced the MDA levels and increased the activities of SOD and CAT, indicating that *L. plantarum* CCFM639 has a direct protective effect against Al-induced oxidative stress and inflammation. These two properties of this strain may not only enable it to protect against Al-induced oxidative stress and inflammation in the intestine, but also against chain effects triggered by oxidative stress and inflammation.

Our research also showed that, although both *L. plantarum* CCFM639 and DFP have good and similar Al sequestration ability in the intestine, the former has a better protective effect of decreasing Al absorption and maintaining gut health than the latter. The better protective effect of *L. plantarum* CCFM639 is due to its enhanced antioxidative and anti-inflammatory activities, allowing it to further protect gut health against Al-induced oxidative stress and inflammation, but DFP only increases Al excretion and thereby decreases intestinal exposure to Al. *L. plantarum* CCFM639 thus has a more comprehensive protective effect than DFP on Al induced gut injuries. Moreover, Lactobacilli are extensively used in the fermented foods and are generally regarded as safe [49]. Therefore, it is possible to develop this strain for its application as functional probiotic and a starter culture in fermented foods, including fermented soymilk, yogurt or it can be supplemented in fruit and vegetable juices to elevate the nutritional value, and increase shelf life with additional benefits of providing protection against Al toxicity.

## 5. Conclusions

This study demonstrates that *L. plantarum* CCFM639 can protect against Al-induced gut injuries, including reducing intestinal epithelial cell cytotoxicity, maintaining the integrity of TJ proteins, modulating inflammation and alleviating oxidative stress in both cell and animal models. These results show that *L. plantarum* CCFM639 can be used in fermented functional foods against oral Al exposure by protecting gut health in daily life.

## Figures and Tables

**Table 1:** The design of cell and animal experiments.

Cell Experiment				Animal Experiment		
Group	Intervention Assay	Therapy Assay		Group	Intervention Assay	
	0–24 h	0–24 h	24–48 h		0–8 Weeks	8–14 Weeks
Control	Medium	Medium	Medium	Control	SM + PW	SM + PW
Al only	Medium + Al	Medium + Al	Medium	Al only	SM + Al	SM + PW
639 only	Medium + 639	Medium	Medium + 639	Al + 639	SM + Al + 639	SM + PW + 639
Al + 639	Medium + Al + 639	Medium + Al	Medium + 639	Al + DFP	SM + Al + DFP	PW + DFP

**Table 2:** The effects of *L. plantarum* CCFM639 on Al-induced alterations of pro-inflammatory cytokines in HT-29 cells.

Group	TNF-α (pg/mL)		IL-1β (pg/mL)		IL-6 (pg/mL)	
	Intervention	Therapy	Intervention	Therapy	Intervention	Therapy
Control	25.12 ± 2.14 ^a^	27.82 ± 1.46 ^a^	6.90 ± 0.45 ^a^	6.12 ± 0.56 ^a^	39.49 ± 0.71 ^a^	40.04 ± 1.18 ^a^
Al only	43.66 ± 2.91 ^b^	49.56 ± 4.37 ^b^	13.23 ± 1.23 ^b^	14.69 ± 1.94 ^b^	52.49 ± 1.58 ^b^	55.82 ± 2.16 ^b^
639 only	23.84 ± 1.78 ^a^	29.50 ± 1.81 ^a^	6.46 ± 0.78 ^a^	6.89 ± 0.23 ^a^	37.80 ± 1.63 ^a^	41.12 ± 1.35 ^a^
Al + 639	30.20 ± 3.73 ^a^	37.08 ± 2.35 ^c^	9.39 ± 0.86 ^c^	11.16 ± 1.05 ^c^	43.66 ± 0.88 ^c^	47.61 ± 1.03 ^c^

Data represent mean ± SEM. The different letters a–c indicate statistically significant changes among four groups (*p* < 0.05).

**Table 3:** The effects of *L. plantarum* CCFM639 on Al-induced alterations of cytokines in small intestine and colon.

Group	Mean Level (pg/mL)		
	TNF-α	IL-1β	IL-6
small intestine			
Control	897.35 ± 51.33 ^a^	45.37 ± 4.33 ^a^	133.23 ± 3.36 ^a^
Al only	1577.37 ± 143.73 ^b^	116.48 ± 8.53 ^b^	169.34 ± 9.53 ^b^
Al + 639	1125.26 ± 63.36 ^c^	83.37 ± 3.07 ^c^	138.71 ± 5.07 ^a^
Al + DFP	1199.36 ± 54.38 ^c^	92.16 ± 4.33 ^d^	160.25 ± 7.13 ^b^
colon			
Control	881.23 ± 25.19 ^A^	44.73 ± 2.19 ^A^	114.35 ± 5.19 ^A^
Al only	1446.78 ± 90.59 ^B^	133.47 ± 5.71 ^B^	148.37 ± 10.71 ^B^
Al + 639	1083.19 ± 59.08 ^C^	66.36 ± 6.32 ^C^	136.74 ± 4.32 ^B^
Al + DFP	1304.37 ± 33.21 ^D^	96.32 ± 3.41 ^D^	145.28 ± 6.41 ^B^

Data represent mean ± SEM. The different letters a–d as well as A–D indicate statistically significant changes among four groups (*p* < 0.05).

## Supplementary Materials

The following are available online at http://www.mdpi.com/2072-6643/8/12/783/s1, Figure S1: Effects of gradient concentration of Al on the viability of HT-29 cells. Values are presented as the mean ± SEM. Figure S2: Effects of *L. plantarum* CCFM639 on Al-induced cytotoxicity in HT-29 cells. (A) Representative histogram of flow cytometric analysis in HT-29 cells. Normal cells (lower left quadrant); early apoptotic cells (lower right quadrant); late apoptotic cells (upper right quadrant); necrotic cells (upper left quadrant). (B) Cell viability in intervention and therapy assays. (C) Percentage of apoptotic cells (the cells in early and late apoptosis) in intervention and therapy assays. Values are presented as the mean ± SEM. The different letters a, b and c indicate statistically significant changes among four groups (*p* < 0.05). Figure S3: Effects of *L. plantarum* CCFM639 on intracellular ROS levels. The ROS was stained with 2,7-dichlorofluorescein diacetate (DCFH-DA; green) and observed with confocal microscopy.
