# Supplementary material for: Potential of Lactobacillus plantarum CCFM639 in Protecting against Aluminum Toxicity Mediated by Intestinal Barrier Function and Oxidative Stress
Source: Nutrients. 2016 Dec 2;8(12):783. doi: 10.3390/nu8120783 (PMC5188438; doi:10.3390/nu8120783)
Supplement: Supplementary file 1 [file nutrients-08-00783-s001.docx]

**Supplementary Materials:** **Potential of *Lactobacillus plantarum* CCFM639 in Protecting against Aluminum Toxicity Mediated by Intestinal Barrier Function and Oxidative Stress**

Leilei Yu, Qixiao Zhai, Fengwei Tian, Xiaoming Liu, Gang Wang, Jianxin Zhao, Jianhua Gong, Hao Zhang, Arjan Narbad and Wei Chen


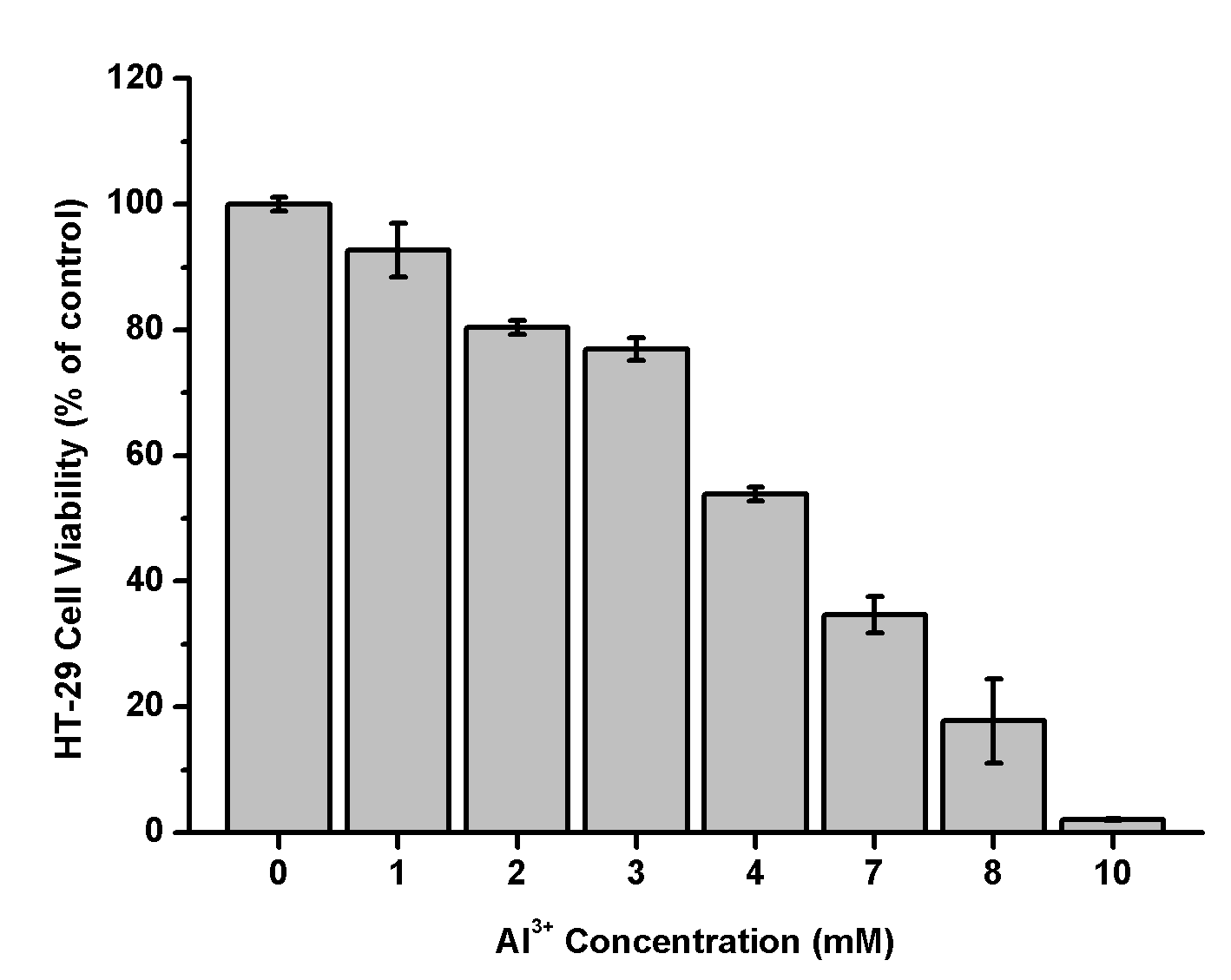


**Figure S1.** Effects of gradient concentration of Al on the viability of HT-29 cells. Values are presented as the mean ± SEM.


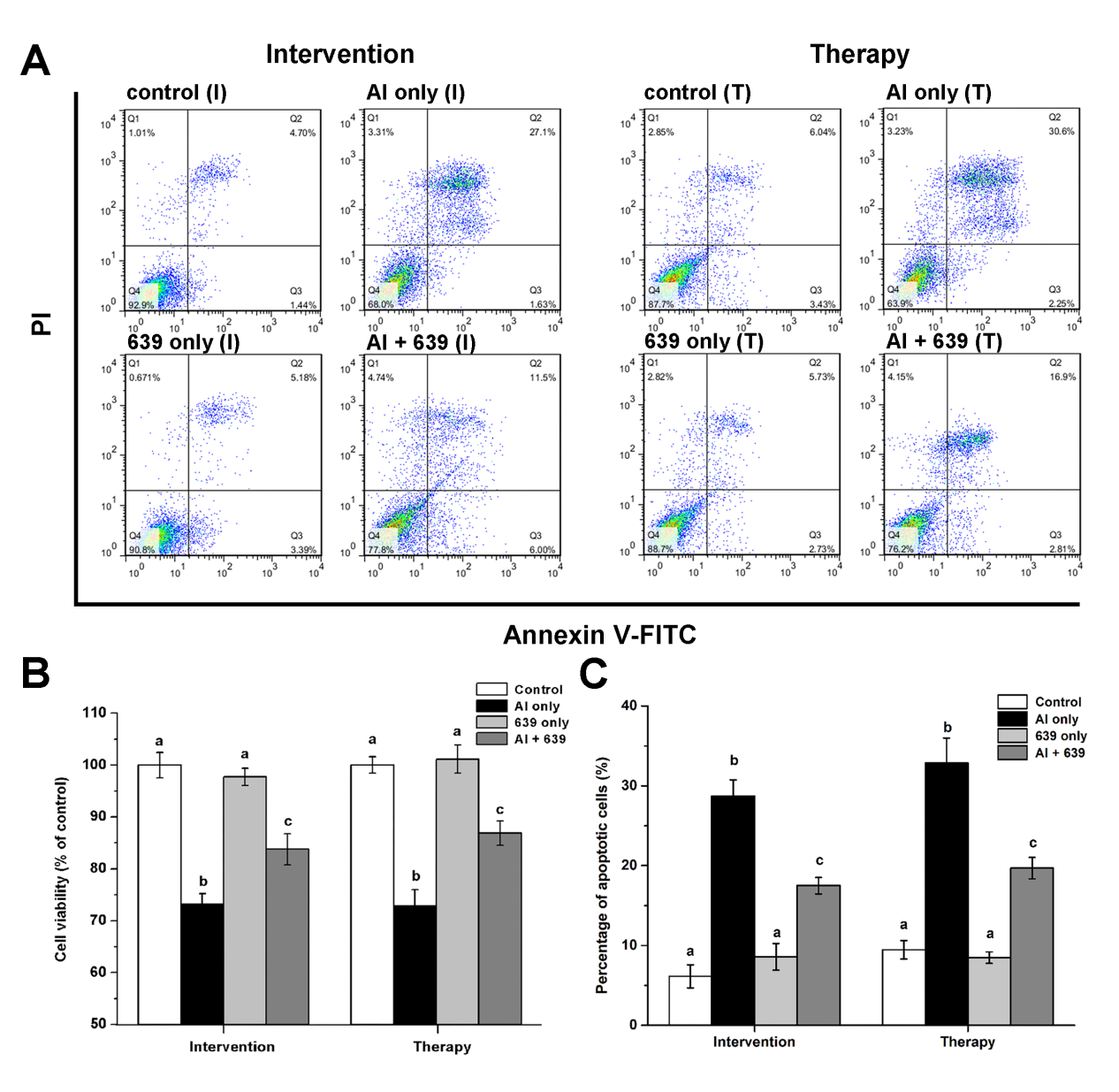


**Figure S2.** Effects of *L. plantarum* CCFM639 on Al-induced cytotoxicity in HT-29 cells. (**A**) Representative histogram of flow cytometric analysis in HT-29 cells. Normal cells (lower left quadrant); early apoptotic cells (lower right quadrant); late apoptotic cells (upper right quadrant); necrotic cells (upper left quadrant); (**B**) Cell viability in intervention and therapy assays; (**C**) Percentage of apoptotic cells (the cells in early and late apoptosis) in intervention and therapy assays. Values are presented as the mean ± SEM. The different letters a, b and c indicate statistically significant changes among four groups (*p* < 0.05).


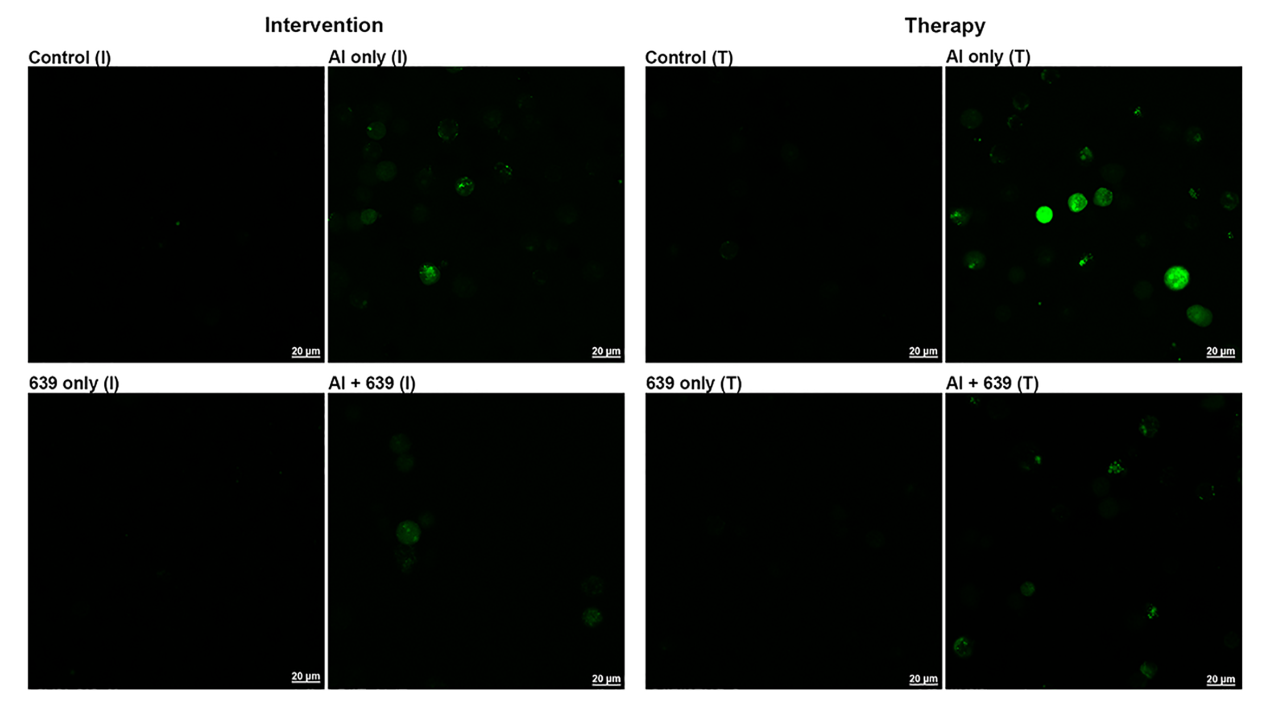


**Figure S3.** Effects of *L. plantarum* CCFM639 on intracellular ROS levels. The ROS was stained with 2,7-dichloroﬂuorescein diacetate (DCFH-DA; green) and observed with confocal microscopy.
